# Response to commentary “The importance of assessing left ventricular longitudinal function in presence of increased afterload”

**DOI:** 10.1186/s13054-024-04886-3

**Published:** 2024-03-29

**Authors:** Hugues de Courson, Alexandre Loiseau, Grégoire Chadefaux, Matthieu Biais

**Affiliations:** 1 Department of Anesthesiology and Critical Care, Bordeaux University Hospital, Place Amélie Raba-Léon, Hôpital Tripode, Troisième Étage Aile 1, 33000 Bordeaux, France; 2 INSERM, BPH, U1219, Univ. Bordeaux, 33000 Bordeaux, France; 3 U1034, Biology of Cardiovascular Diseases, INSERM, 33600 Pessac, France; 4 University of Bordeaux, Bordeaux, France

Dear Editor,

We very much appreciate Dr. Santonocito's thoughtful comments on our research, titled Myocardial dysfunction assessed by speckle-tracking in good-grade subarachnoid hemorrhage patients (WFNS 1–2): a prospective observational study [1].

The authors raise two valuable points:

*Threshold for defining left ventricular damage* We agree that the chosen threshold may have been too high for our specific population. We addressed this issue in our paper by presenting and discussing results for a lower threshold of − 17%. However, it's important to note that the research questioning this common threshold in critical care patients wasn't published at the time our protocol design and clinical trial registration (NCT03761654) were finalized.

*Accounting for high afterload* We agree that high afterload could influence the results. Note that the S' wave results presented were for the right ventricle. However, we were able to re-analyse the ultrasound images to obtain the value of the lateral S' wave at the mitral level. So, we performed, as asked, further analysis to assess the concordance and correlation between global longitudinal strain and mitral S' wave, providing a more comprehensive picture of left ventricular function in this context.The correlation between S' wave and SLG was very low and not statistically significant (r = − 0.023; *p* = 0.875). Using a threshold of 6.8 according to the work of *Park *et al. [2], the concordance rate between GLS and S' wave was 45% for a pathological GLS threshold of − 20% and 79% for a pathological GLS threshold of − 17% (Fig. 1).

**Fig. 1:**
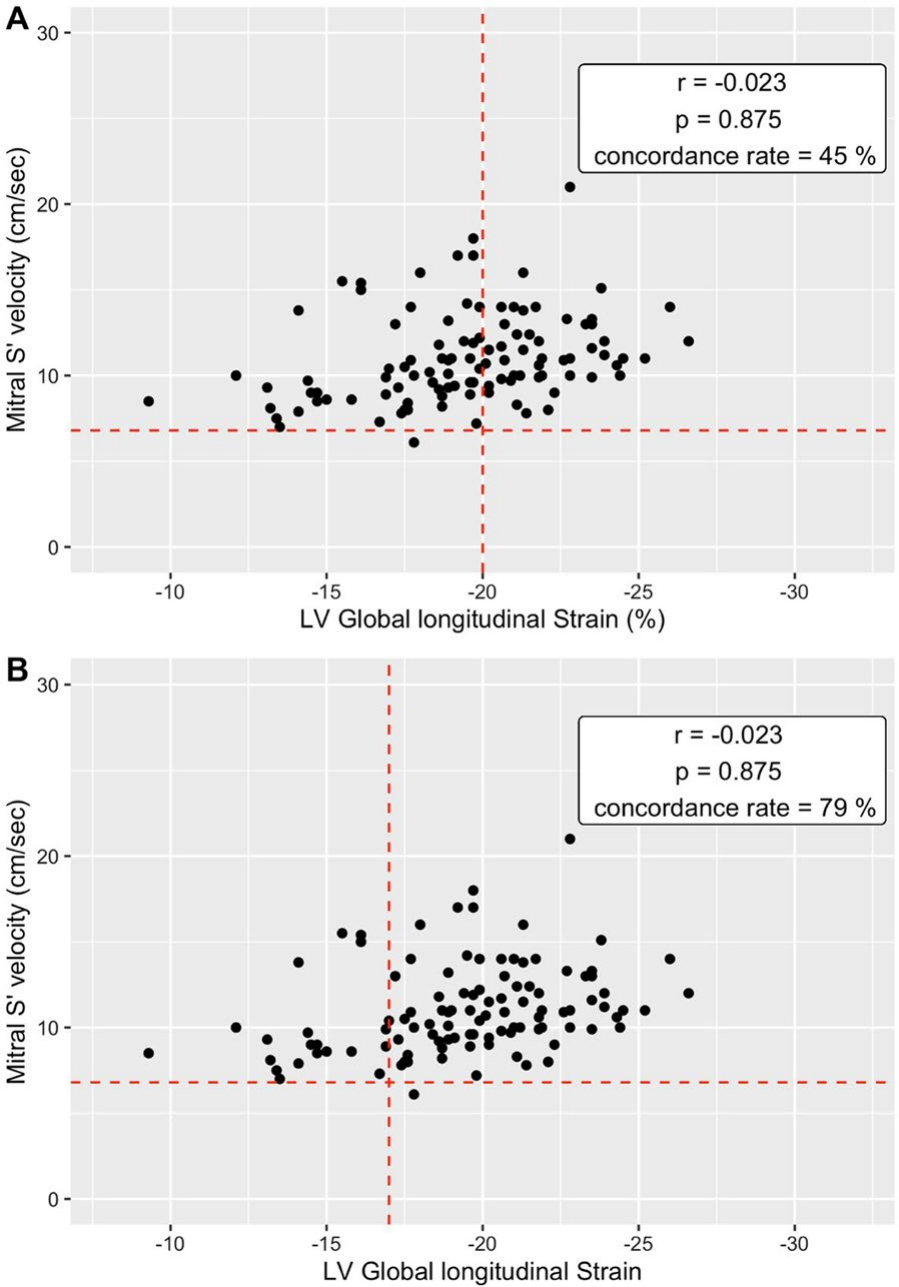
Relationship between left ventricular ejection fraction (LVEF) and Mitral S’ velocity. **A** Strain threshold of − 20. **B** Strain threshold of − 17

These results therefore suggest that mitral S' wave analysis is not a better surrogate for GLS in this population.

## Data Availability

All data generated or analyzed during this study are included in this published article.

## Abbreviations

GLS: Global longitudinal strain
WFNS: World Federation of Neurologic Surgeons
